# Awareness, Attitude, and Current Practices Toward Influenza Vaccination Among Physicians in India: A Multicenter, Cross-Sectional Study

**DOI:** 10.3389/fpubh.2021.642636

**Published:** 2021-08-23

**Authors:** Agam Vora, Ashfaque Shaikh

**Affiliations:** ^1^Vora Clinic, Mumbai, India; ^2^Abbott India Limited, Mumbai, India

**Keywords:** attitude, awareness, current practices, India, influenza, influenza vaccination, healthcare providers

## Abstract

**Background:** Although annual influenza vaccination is recommended for healthcare providers (HCPs), vaccination rate among HCPs in India is generally low. This cross-sectional survey was conducted to evaluate physicians' awareness, attitude, and current practices toward influenza vaccination in high-risk groups in India.

**Methods:** The survey was performed in June–July 2020, wherein consulting physicians, pulmonologists, diabetologists, obstetricians/gynecologists, or cardiologists across 14 cities completed a 39-item questionnaire consisting of 3 sections, one each on awareness, attitude, and practice patterns. Descriptive statistics were used to summarize the study results. Statistical analysis was performed for comparison of subgroups by physician specialty, city of practice (metro/non-metro), and zone of practice (north/south/east/west). Level of statistical significance was set at *p* < 0.05.

**Results:** In all, 780 physicians completed the survey. Of these, 3.97, 53.08, and 42.95% had high, medium, and low level of awareness about influenza/influenza vaccination, respectively. Statistically significant (*p* < 0.05) between-group differences were found by physician specialty and zone of practice. In terms of attitude toward vaccination of high-risk group subjects, only 0.9% physicians were “extremely concerned,” while the majority (92.56%) were “quite concerned” and 6.54% were a “little concerned,” with no reported significant differences between different subgroups. With regard to practice patterns, 82.82% of physicians offered influenza vaccines to their patients, 32.69% vaccinated 10–25% of patients per month, and 38.85% required and offered the vaccine to their office staff. Physicians' reasons for not prescribing influenza vaccines to patients included fear of side effects (16.54%), cost (15.64%), lack of awareness about availability (15.38%), absence of belief that it is beneficial (14.36%), history of side effects (13.46%), and patients' fear of needles (11.28%).

**Conclusion:** These findings suggest the need to implement educational strategies among physicians to enhance their awareness about influenza vaccination and improve their attitudes and current practices toward influenza vaccination especially in high-risk groups in India.

## Introduction

Influenza is an acute respiratory infectious disease caused by the influenza virus (1). Influenza causes 3–5 million cases of severe respiratory tract infections and 3–6 million respiratory deaths per year globally (2, 3). In India, influenza cases have increased 5-fold from 2012 (5,044) to 2019 (28,798) (4). Influenza is also one of the major causes of high morbidity and mortality in high-risk groups, including healthcare providers (HCPs), pregnant women, children aged from 6 months to 5 years of age, elderly individuals (aged more than 65 years), and individuals with chronic medical conditions (1). Hence, the Ministry of Health and Family Welfare (Government of India), World Health Organization, and the Center for Disease Control (CDC) have recommended annual influenza vaccination for all the high-risk groups given their susceptibility to influenza-related complications and mortality (5).

HCPs have an additional risk of acquiring influenza infection as compared to the general population because of their proximity to patients (6). Moreover, infected or unvaccinated HCPs can transmit the infection to patients and clinical staff. The transmission of influenza virus from HCPs to patients is often cited as the principal source of nosocomial influenza outbreaks (7–9). The risk of transmission in high-risk healthcare settings, like emergency departments, critical care units, employee health clinics, and infection control departments, further imposes detrimental outcomes (10). Hence, HCPs should be vaccinated to protect themselves and avoid subsequent absenteeism issues to continue serving patients. Previous studies have shown a direct correlation between HCP vaccination and increased patient protection against influenza infection in acute and long-term care settings (11, 12). Moreover, HCP absenteeism was found to result in untoward effects on patient health, particularly in intensive care units (13), and a considerable decline in HCP absenteeism from healthcare services was seen after HCP vaccination (14).

Despite the availability of a well-tolerated and effective vaccine for influenza, data indicates that the personal vaccination coverage among HCPs is still low (15–18). As physicians play a pivotal role in increasing the vaccination rate across the public sector, it is very important to enhance their awareness, perception, and current practices toward influenza vaccination. However, no such study has been conducted to determine the awareness, attitudes, and current practices of physicians toward influenza vaccination in India. The present cross-sectional survey was conducted to evaluate the current knowledge, attitude, and practices of Indian physicians toward influenza vaccination in high-risk groups with the aim to identify knowledge gaps across different physician specialties and to aid in planning mitigation strategies to bridge these gaps.

## Methods

### Survey Design

A list of physicians across different specialties (obstetrics/gynecology, pulmonology, diabetology, cardiology, and consultant physicians) and different zones of India was prepared before the start of the survey to maintain a balanced distribution of survey sample. All the physicians were contacted telephonically, the purpose and rationale of the survey was explained, and their willingness to participate in the survey was sought. Once the physician agreed to participate in the survey, consent was taken electronically, and the weblink for filling the survey form was shared *via* an email. The data collection lasted from June to July 2020.

The study planned to recruit at least 780 physicians (156 each from different specialties) across 14 cities of India [north (4 cities): Delhi, Lucknow, Chandigarh, and Jaipur; south (4 cities): Hyderabad, Bangalore, Chennai, and Cochin; east (2 cities): Kolkata and Guwahati; and west (4 cities): Mumbai, Ahmedabad, Pune, and Indore]. Due to non-interventional nature of the survey, no formal sample size was calculated. A total of 786 physicians were contacted to fill the survey forms.

This survey was conducted in conformance with the principles of the Declaration of Helsinki, International Conference on Harmonization-Good Clinical Practice (GCP) guidelines, Indian Council of Medical Research and Indian GCP guidelines, and the study protocol. In accordance with local legislation and national guidelines, ethical approval by an independent ethics review board was not required because this survey did not involve any intervention or direct participation of a patient. However, informed consent was obtained from all the participating physicians, and physician confidentiality and anonymity were maintained throughout the study conduct.

### Eligibility Criteria

Physicians who had >5 years of clinical experience and who had been treating high-risk patients with an increased risk of influenza-associated complications like pneumonia, myocarditis, encephalitis, myositis, worsening congestive heart failure, worsening asthma, and diabetes were included in the survey. High-risk groups for influenza include the categories of people that develop severe symptoms leading to hospitalization and, in some cases death, as per the Centers for Disease Control and Prevention guidelines.

### Survey Questionnaire

The survey was designed based on previously published literature (17, 19–23) and consisted of 39 questions categorized in to 3 sections: (1) section 1 on physician awareness with 26 questions; (2) section 2 on physician attitude with 9 questions; and (3) section 3 on physicians' current practices with 4 questions (Supplementary Table 1). The cutoff scores for physician awareness and attitude about influenza and influenza vaccination were established a priori.

Awareness was categorized as low (≤ 16 points), medium (17–21 points), or high (≥22 points) based on the number of correct responses. Each correct answer received 1 point and incorrect or unsure responses received no points. Physicians' attitude was categorized as extremely concerned (if agreement was evident for all 9 questions), quite concerned (if agreement was evident for 4–8 questions), little concerned (if agreement was evident for 1–3 questions), or not concerned (if there was no agreement). Responses were rated on a 5-point Likert scale where score 5 = strongly agree, score 4 = agree, score 3 = do not know, score 2 = disagree, and score 1 = strongly disagree. Agreement was defined as strongly agree/agree/correct response to positive statements and strongly disagree/disagree/incorrect response to negative statements.

Each physician was assigned a unique respondent identification number to facilitate back-tracing, if needed, to verify/clarify the responses.

Demographic information such as age, gender, specialty, and total years of job experience of the physicians was also recorded.

### Assessments

The percentage of physicians with high, medium, or low level of awareness about influenza and influenza vaccination was assessed. In addition, percentage of physicians with little, quite, or extremely concerned attitude toward influenza vaccination of high-risk groups; percentage of physicians who offered influenza vaccine in their clinical practice; percentage of patients vaccinated by a physician in a month; percentage of physicians who required and offered the influenza vaccine to their office staff; and the reasons, in descending order, for not prescribing influenza vaccine to patients were also assessed.

### Statistical Analyses

All physicians who participated in the survey constituted the analysis population. Quantum statistical cross tabulation package (Quantum Software) was used to run the data tables where the data was cross tabulated to quantitatively analyze the relationship between multiple variables. Categorical data were summarized as number (percentage) of patients. Statistical comparisons were made by type of specialty (consulting physicians, pulmonologists, diabetologists, obstetricians and gynecologists, or cardiologists), type of city of practice (metro or non-metro), and zone of practice (north, south, east, or west). The independent sample *t*-test was used to compare differences in continuous variables, and the chi-square test was used to compare distribution of proportion in different categorical variables. The level of statistical significance was set at *p* < 0.05.

## Results

### Physician Demographics

A total of 786 physicians were contacted to fill the survey forms. Of these, 6 physicians provided incomplete forms and were excluded from the analysis. Of 780 physicians included in the analysis, 156 each were consulting physicians, pulmonologists, diabetologists, obstetricians and gynecologists, and cardiologists. The mean age of participating physicians was 46.35 years, with experience level ranging from 5 to 50 years. The physicians were predominantly male (76.79 vs. 23.21%) and from non-metro cities (56.92 vs. 43.08%) (Table 1).

**Table 1:** Physician demographics.

***n* (%)**	**Overall *N* = 780**	**Specialty**				
		**CPs**	**Pulmonologists**	**Diabetologists**	**OBGYN**	**Cardiologists**
		**(*n* = 156)**	**(*n* = 156)**	**(*n* = 156)**	**(*n* = 156)**	**(*n* = 156)**
**Sig (95%CL)**		**a**	**b**	**c**	**d**	**e**
**Age (years)**						
<30	6 (0.77)	1 (0.64)	3 (1.92)	0	1 (0.64)	1 (0.64)
31–40	208 (26.67)	36 (23.08)	46 (29.49)	41 (26.28)	46 (29.49)	39 (25.00)
41–50	353 (45.26)	58 (37.18)	^*^76 (48.72) (a)	72 (46.15)	71 (45.51)	^*^76 (48.72) (a)
51–60	176 (22.56)	^*^47 (30.13) (b)	25 (16.02)	39 (25.00)	32 (20.51)	33 (21.15)
60+	37 (4.74)	^*^14 (8.97) (c)	6 (3.85)	4 (2.57)	6 (3.85)	7 (4.49)
Mean (years)	46.35	48.03 (bd)	44.97	46.42	45.68	46.67
**Gender**						
Male	599 (76.79)	^*^145 (92.95) (d)	^*^139 (89.10) (d)	^*^137 (87.82) (d)	34 (21.79)	^*^144 (92.31) (d)
Female	181 (23.21)	11 (7.05)	17 (10.90)	19 (12.18)	^*^122 (78.21) (abce)	12 (7.69)
**Total experience (years)**						
5–10	146 (18.72)	27 (17.31)	39 (25.00)	23 (14.74)	26 (16.67)	31 (19.87)
11–15	222 (28.46)	41 (26.28)	41 (26.28)	44 (28.21)	52 (33.33)	44 (28.21)
16–20	163 (20.9)	26 (16.67)	36 (23.08)	35 (22.44)	30 (19.23)	36 (23.08)
21–25	127 (16.28)	28 (17.95)	23 (14.74)	30 (19.23)	23 (14.74)	23 (14.74)
26–30	72 (9.23)	14 (8.97)	10 (6.41)	17 (10.9)	17 (10.9)	14 (8.97)
31–35	32 (4.1)	10 (6.41)	6 (3.85)	6 (3.85)	5 (3.21)	5 (3.21)
36–40	14 (1.79)	8 (5.13)	1 (0.64)	1 (0.64)	2 (1.28)	2 (1.28)
41–45	3 (0.38)	1 (0.64)	0	0	1 (0.64)	1 (0.64)
46–50	1 (0.13)	1 (0.64)	0	0	0	0
***n*** **(%)**	**City of practice**		**Zone of practice**			
	**Metro (** ***n*** **= 336)**	**Non-metro (** ***n*** **= 444)**	**North (** ***n*** **= 223)**	**South (** ***n*** **= 224)**	**East (** ***n*** **= 112)**	**West (** ***n*** **= 221)**
**Sig (95%CL)**	**f**	**g**	**h**	**i**	**j**	**k**
**Age (years)**						
<30	3 (0.89)	3 (0.68)	0	1 (0.45)	1 (0.89)	^*^4 (1.81) (h)
31–40	98 (29.17)	110 (24.77)	36 (16.14)	^*^58 (25.89) (h)	^*^34 (30.36) (h)	^*^80 (36.20) (hi)
41–50	147 (43.75)	206 (46.40)	^*^107 (47.98) (jk)	^*^128 (57.14) (jk)	36 (32.14)	82 (37.10)
51–60	70 (20.83)	106 (23.87)	^*^61 (27.36) (i)	36 (16.07)	36 (32.14) (ik)	43 (19.46)
60+	18 (5.36)	19 (4.28)	^*^19 (8.52) (i)	1 (0.45)	5 (4.47) (i)	^*^12 (5.43) (i)
Mean (years)	46.03	46.6	^*^49.09 (ijk)	45.15	46.7 (k)	44.64
**Gender**						
Male	251 (74.70)	348 (78.38)	171 (76.68)	165 (73.66)	91 (81.25)	172 (77.83)
Female	85 (25.30)	96 (21.62)	52 (23.32)	59 (26.34)	21 (18.75)	49 (22.17)
**Specialty**						
CPs	68 (20.24)	88 (19.82)	45 (20.18)	45 (20.09)	22 (19.64)	44 (19.91)
Pulmonologists	66 (19.64)	90 (20.27)	45 (20.18)	45 (20.09)	22 (19.64)	44 (19.91)
Diabetologists	67 (19.94)	89 (20.04)	45 (20.18)	44 (19.64)	23 (20.54)	44 (19.91)
OGBYN	67 (19.94)	89 (20.05)	44 (19.73)	45 (20.09)	23 (20.54)	44 (19.91)
Cardiologists	68 (20.24)	88 (19.82)	44 (19.73)	45 (20.09)	22 (19.64)	45 (20.36)
**Total experience (years)**						
5–10	68 (20.24)	78 (17.57)	27 (12.11)	43 (19.2)	27 (24.11)	49 (22.17)
11–15	99 (29.46)	123 (27.7)	56 (25.11)	83 (37.05)	29 (25.89)	54 (24.43)
16–20	61 (18.15)	102 (22.97)	52 (23.32)	55 (24.55)	18 (16.07)	38 (17.19)
21–25	56 (16.67)	71 (15.99)	43 (19.28)	33 (14.73)	16 (14.29)	35 (15.84)
26–30	34 (10.12)	38 (8.56)	24 (10.76)	6 (2.68)	18 (16.07)	24 (10.86)
31–35	13 (3.87)	19 (4.28)	16 (7.17)	4 (1.79)	3 (2.68)	9 (4.07)
36–40	5 (1.49)	9 (2.03)	5 (2.24)	0	0	9 (4.07)
41–45	0	3 (0.68)	0	0	0	3 (1.36)
46–50	0	1 (0.23)	0	0	1 (0.89)	0

*CL, confidence level; CPs, consulting physicians; OBGYN, obstetricians and gynecologists; Sig, significance*.

*Each category was assigned alphabets a–k, and if a category was significantly different from any other category, it was marked by the respective category alphabet in parentheses*.

* *Denotes statistically significantly higher proportion than the other respective categories mentioned in parentheses. P-values < 0.05 was considered as statistically significant*.

### Physicians' Awareness Toward Influenza and Influenza Vaccination

Majority of the physicians believed that influenza is more severe than common cold (85.64%), and the transmission is primarily by coughing and sneezing (85.13%) and not by contact with blood and body fluids (44.87%). Some physicians had the wrong notion that people with influenza can transmit the infection only after their symptoms appear (57.05%). Most (83.08%) of the physicians were aware of the disease signs and symptoms, but 66.5% of physicians had also mentioned that people with influenza commonly experience nausea and vomiting or diarrhea. A total of 57.82% of physicians stated that the symptoms do not typically appear in 8–10 days post-exposure to influenza. Around 70.38% of the physicians were aware that all the high-risk groups have a higher susceptibility to influenza infection. Regarding awareness toward influenza vaccination, most physicians believed that not everyone in the general public is familiar with influenza vaccination (88.21%) and considered word of mouth by HCPs (74.23%), in-clinic patient education (65%), and public awareness campaigns (63.85%) as effective ways of publicizing the influenza vaccine. Physicians were aware that influenza vaccine could be live or attenuated (83.46%), but 64.62% of the physicians had the misconception that the inactivated influenza vaccines contain live viruses that may cause influenza and that the vaccine protects a person from infection for 1–2 years (59.49%) instead of 6–8 months. Most (76.03%) of the physicians were aware of the differences between trivalent influenza vaccines (TIVs) and quadrivalent influenza vaccines (QIVs), and 86.79% were aware that QIVs offer broader protection over TIVs. Around 67.56% of physicians were aware of the differences between subunit and split influenza vaccines, and 82.69% of physicians were aware that the subunit vaccines have a lower reactogenicity than the split vaccines. The influenza vaccine was reported as tolerable by 83.46% of physicians, and 84.1% of physicians were aware that the vaccine efficacy might be reduced if there was a mismatch of virus strains. The physicians were aware of the guideline(s) on preventive care for influenza (55.77%) (of which the CDC guideline was the most common guideline stated by physicians [37%]). In all, 82.05% of physicians were aware about the CDC recommendations on influenza shots for HCPs, 84.62% were aware that the vaccine needs to be taken annually, and 78.97% were aware that the appropriate time for vaccination is before the start of flu season. Physicians were also aware that they are at risk of getting influenza infection and should be vaccinated annually (87.44%) and that they could spread the disease to their patients (83.97%; Table 2). Overall, the level of awareness about influenza and influenza vaccination was medium in 53.08% physicians, low in 42.95% physicians, and high in only 3.97% of physicians (Figure 1). There were no statistically significant differences for proportion of physicians with medium or high awareness across different specialties, type of city of practice, or zone of practice. However, a significant difference was observed for proportion of physicians with low awareness by zone of practice east vs. north, south, or west (*p* < 0.05).

**Table 2:** Physician awareness toward influenza and influenza vaccination.

**Correct response, %**	**Overall** ***N* = 780**	**Specialty**				
		**CPs** **(*n* = 156)**	**Pulmonologists** **(*n* = 156)**	**Diabetologists** **(*n* = 156)**	**OBGYN** **(*n* = 156)**	**Cardiologists** **(*n* = 156)**
**Sig (95%CL)**		**a**	**b**	**c**	**d**	**e**
**Influenza is more serious than a “common cold”**						
	85.64	86.54	^#^88.46 (c)	80.13	85.26	87.82
**The signs and symptoms of influenza include fever, headache, sore throat, cough, nasal congestion, and aches and pains**						
	83.08	84.62	82.05	80.13	83.97	84.62
**Adults with influenza do not commonly experience nausea and vomiting or diarrhea**						
	31.6	35.90	32.69	27.56	33.97	28.21
**Symptoms do not typically appear 8–10 days after a person is exposed to influenza**						
	57.82	53.21	55.13	57.05	61.54	^#^62.18 (a)
**Influenza is transmitted primarily by coughing and sneezing**						
	85.13	80.13	87.18	85.90	87.18	85.26
**Influenza is not transmitted primarily by contact with blood and body fluids**						
	44.87	39.10	46.15	39.10	^#^51.28 (ac)	48.72
**People with influenza cannot transmit the infection only after their symptoms appear**						
	37.56	34.62	39.74	34.62	33.97	44.87
**Not everyone in general public is familiar with influenza vaccination**						
	88.21	89.10	90.38	84.62	88.46	88.46
**The most effective way of publicizing influenza vaccination are:**						
HCP word of mouth						
	74.23	75.64	75.64	75.64	73.72	70.51
In-clinic patient education/counseling						
	65.00	66.67	68.59	67.95	61.90	60.26
**Influenza vaccines can be live or inactivated**						
	83.46	^#^87.82 (d)	83.97	82.05	79.49	83.97
**In case of mismatch of virus strains, the influenza vaccine efficacy may be reduced**						
	84.10	83.97	84.62	82.05	83.33	86.61
**I believe influenza vaccine is tolerable**						
	83.46	85.26	87.18	85.26	79.49	80.13
**The inactivated influenza vaccine does not contain live viruses that may cause some people to get influenza**						
	27.95	21.79	29.49	26.28	28.85	^#^33.33 (a)
**Influenza vaccine should be administered every year**						
	76.92	75.64	74.36	^#^83.98 (bd)	73.72	76.92
**Influenza vaccine can protect for 6–8 months**						
	38.08	37.18	37.18	38.46	37.18	40.38
**The appropriate time to give influenza vaccine is before flu season starts**						
	78.97	76.92	82.05	77.57	77.56	80.77
**Pregnancy and 2 weeks post-partum, children <2 years, elderly >65 years are the high-risk groups associated with influenza**						
	70.38	66.67	70.51	70.51	71.79	72.44
**Influenza vaccine needs to be taken on an annual basis**						
	84.62	83.98	86.54	82.05	81.41	89.10
**I feel that I am at risk to get influenza and should get vaccinated annually**						
	87.44	85.26	87.82	88.46	86.54	89.10
**Physicians can spread influenza to their patients**						
	83.97	81.41	86.54	83.33	82.69	85.90
**Guideline exists on preventive care for influenza**						
	55.77	56.41	51.92	^#^63.46 (bd)	51.92	55.13
**Center for Disease Control recommends that healthcare practitioners should receive the influenza shot**						
	82.05	84.62	84.62	79.49	81.41	80.13
**There is a difference between trivalent and quadrivalent influenza vaccines**						
	76.03	77.56	^#^80.77 (e)	71.79	78.85	71.15
**A quadrivalent influenza vaccine offers broad protection over a trivalent flu vaccine**						
	86.79	87.18	89.10	84.62	89.10	83.97
**There is a difference between subunit and split influenza vaccines**						
	67.56	^#^69.87 (d)	^#^73.72 (d)	66.03	57.05	^#^71.15 (d)
**A subunit flu vaccine is less reactogenic**						
	82.69	82.05	85.90	83.97	83.33	78.21
**Correct response, %**	**City of practice**		**Zone of practice**			
	**Metro**	**Non-metro**	**North**	**South**	**East**	**West**
	**(** ***n*** **= 336)**	**(** ***n*** **= 444)**	**(** ***n*** **= 223)**	**(** ***n*** **= 224)**	**(** ***n*** **= 112)**	**(** ***n*** **= 221)**
**Sig (95%CL)**	**f**	**g**	**h**	**i**	**j**	**k**
**Influenza is more serious than a “common cold”**						
	81.85	^#^88.51 (f)	85.20	86.16	79.46	^#^88.69 (j)
**The signs and symptoms of influenza include fever, headache, sore throat, cough, nasal congestion, and aches and pains**						
	85.42	81.31	81.61	83.04	83.93	84.16
**Adults with influenza do not commonly experience nausea and vomiting or diarrhea**						
	^#^35.71 (g)	28.60	^#^38.57 (ik)	28.57	^#^38.39 (k)	24.43
**Symptoms do not typically appear 8–10 days after a person is exposed to influenza**						
	59.29	56.76	42.15	55.80	62.50	^#^73.30 (hij)
**Influenza is transmitted primarily by coughing and sneezing**						
	84.82	85.58	87.00	83.48	85.71	84.62
**Influenza is not transmitted primarily by contact with blood and body fluids**						
	^#^65.77 (g)	29.05	50.22 (k)	^#^62.05 (hk)	^#^64.29 (hk)	12.22
**People with influenza cannot transmit the infection only after their symptoms appear**						
	^#^56.85 (g)	22.97	37.67 (k)	^#^52.23 (hk)	^#^50.89 (hk)	15.84
**Not everyone in general public is familiar with influenza vaccination**						
	89.88	86.94	87.45	88.39	84.82	90.50
**The most effective way of publicizing influenza vaccination are:**						
HCP word of mouth						
	^#^85.12 (g)	65.99	64.57	^#^87.95 (hjk)	^#^78.57 (hk)	67.87
In-clinic patient education/counseling						
	^#^77.38 (g)	55.63	38.12	^#^87.95 (hjk)	^#^79.46 (hk)	^#^61.54 (h)
**Influenza vaccines can be live or inactivated**						
	80.95	85.36	^#^89.24 (i)	70.98	^#^87.50 (i)	^#^88.24 (i)
**In case of mismatch of virus strains, the influenza vaccine efficacy may be reduced**						
	86.61	82.21	^#^87.00 (i)	79.46	80.36	^#^87.78 (i)
**I believe influenza vaccine is tolerable**						
	82.14	84.46	^#^89.24 (i)	75.00	^#^83.04 (i)	^#^86.43 (i)
**The inactivated influenza vaccine does not contain live viruses that may cause some people to get influenza**						
	^#^41.67 (g)	17.57	^#^31.84 (k)	^#^37.95 (k)	^#^30.36 (k)	12.67
**Influenza vaccine should be administered every year**						
	^#^83.63 (g)	71.85	^#^82.06 (k)	^#^79.02 (k)	78.57	68.78
**Influenza vaccine can protect for 6–8 months**						
	^#^45.53 (g)	32.43	^#^40.36 (k)	^#^46.88 (jk)	30.36	30.77
**The appropriate time to give influenza vaccine is before flu season starts**						
	80.95	77.48	81.62	75.89	82.14	77.83
**Pregnancy and 2 weeks post-partum, children <2 years, elderly >65 years are the high-risk groups associated with influenza**						
	^#^75.89 (g)	66.22	70.85	71.43	74.11	66.97
**Influenza vaccine needs to be taken on an annual basis**						
	86.01	83.56	82.06	84.37	83.03	88.24
**I feel that I am at risk to get influenza and should get vaccinated annually**						
	86.61	88.06	88.34	86.61	84.82	88.69
**Physicians can spread influenza to their patients**						
	81.85	85.59	84.30	78.13	86.61	^#^88.24 (i)
**Guideline exists on preventive care for influenza**						
	^#^71.73 (g)	43.69	28.70	^#^54.02 (h)	^#^75.89 (hi)	^#^74.66 (hi)
**Center for Disease Control recommends that healthcare practitioners should receive the influenza shot**						
	77.68	^#^85.36 (f)	^#^83.41 (i)	71.88	^#^87.50 (i)	^#^88.24 (i)
**There is a difference between trivalent and quadrivalent influenza vaccines**						
	^#^84.23 (g)	69.82	57.40	^#^77.68 (h)	^#^88.39 (hi)	^#^86.88 (hi)
**A quadrivalent influenza vaccine offers broad protection over a trivalent flu vaccine**						
	^#^91.96 (g)	82.88	83.41	87.50	89.29	88.24
**There is a difference between subunit and split influenza vaccines**						
	^#^73.51 (g)	63.06	43.95	^#^76.79 (h)	^#^69.64 (h)	^#^81.00 (hj)
**A subunit flu vaccine is less reactogenic**						
	83.04	82.43	83.86	79.02	76.79	^#^88.24 (ij)

*CL, confidence level; CPs, consulting physicians; HCP, healthcare physician; OBGYN, obstetricians and gynecologists; Sig, significance*.

*Each category was assigned alphabets a–k, and if a category was significantly different from any other category, it was marked by the respective category alphabet in parentheses*.

# *Denotes statistically significantly higher proportion than the other respective categories mentioned in parenthesis. P-values < 0.05 was considered as statistically significant*.

**Figure 1:**
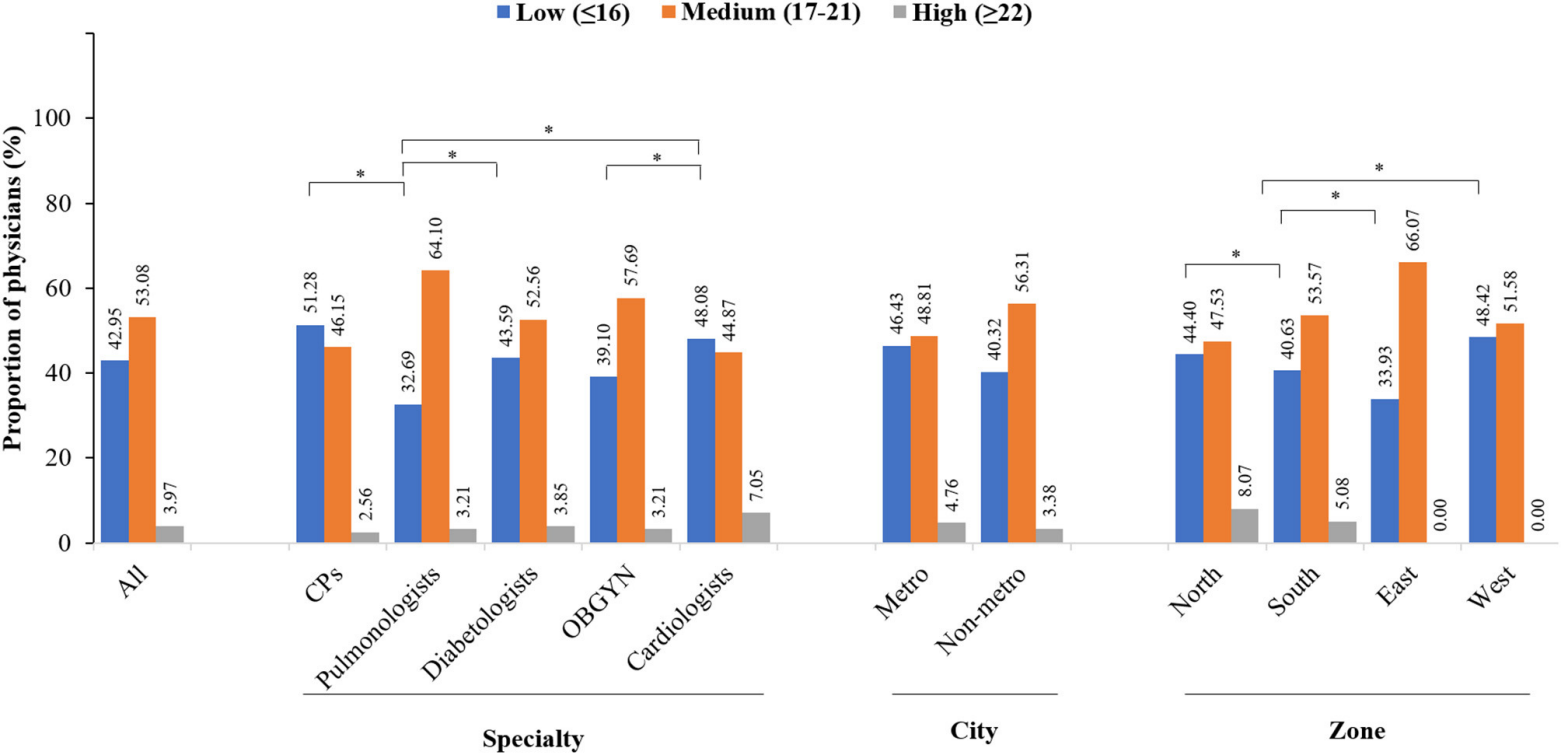
Overall awareness of physicians. *Indicates *P* < 0.05. CPs, consulting physician; OBGYN, obstetricians and gynecologists.

### Physicians' Attitude Toward Influenza Vaccination

Most physicians believed that they could play a key role in vaccination of their patients (86.28%), and influenza vaccine administration should be a part of their routine medical practice (80.64%). They held a strong attitude that vaccination prevents severe complications among the high-risk patients (82.95%) and mandatory vaccination of HCPs would avoid the spread of influenza (85.9%). However, only 57.95% of the physicians got themselves vaccinated against influenza in the last 6–12 months. Around 81.92% of physicians stated that they or their staff would be interested in participating in training related to influenza vaccination, but 62.05% of physicians or their staff had actually participated in training or continuing medical education (CME) related to influenza vaccine in the past 12 months. Majority of the physicians had an attitude that side effects and safety concerns hindered them from vaccinating their patients (76.79%) and that people avoided taking influenza vaccine due to its high cost (76.28%; Table 3).

**Table 3:** Physician attitude toward influenza vaccination.

**Correct response, %**	**Overall** ***N* = 780**	**Specialty**				
		**CPs**	**Pulmonologists**	**Diabetologists**	**OBGYN**	**Cardiologists**
		**(*n* = 156)**	**(*n* = 156)**	**(*n* = 156)**	**(*n* = 156)**	**(*n* = 156)**
**Sig (95%CL)**		**a**	**b**	**c**	**d**	**e**
**A physician should vaccinate oneself against influenza in past 6–12 months**						
	57.95	58.33	59.61	57.69	53.84	60.25
**Influenza vaccine should be part of our routine medical practice**						
	80.64	82.05	83.97	80.77	79.49	76.92
**Influenza vaccines is costly and that is why it is not purchased normally**						
	76.28	85.90	76.92	74.36	75.00	69.23
**Side effects and safety concerns do not hinder physicians to get people vaccinated for influenza**						
	18.72	13.46	20.51	19.87	21.79	17.95
**Influenza vaccine prevents serious complications among patients with high-risk**						
	82.95	83.97	84.61	81.41	82.69	82.05
**I believe that mandatory flu vaccination of healthcare professionals will prevent influenza spread**						
	85.90	88.46	86.54	86.54	85.26	82.69
**I believe I can play a key role in the vaccination of my patients**						
	86.28	87.18	85.26	82.69	89.10	87.18
**Myself or my staff have participated in any training or continuing education related to the influenza vaccine in the past 12 months**						
	62.31	60.90	64.10	64.10	62.18	60.26
**Myself or my staff would be interested in participating in the trainings related to influenza vaccine**						
	81.92	80.77	84.62	80.13	82.05	82.05
**Correct response, %**	**City of practice**		**Zone of practice**			
	**Metro**	**Non-metro**	**North**	**South**	**East**	**West**
	***(n*** **= 336)**	**(** ***n*** **= 444)**	**(** ***n*** **= 223)**	**(** ***n*** **= 224)**	**(** ***n*** **= 112)**	**(** ***n*** **= 221)**
**Sig (95%CL)**	**f**	**g**	**h**	**i**	**j**	**k**
**A physician should vaccinate oneself against influenza in past 6–12 months**						
	45.24	^#^67.57 (f)	^#^64.12 (i)	33.48	^#^54.46 (i)	^#^78.28 (hij)
**Influenza vaccine should be part of our routine medical practice**						
	^#^83.93 (g)	78.15	80.27	73.21	82.14	^#^87.78 (hi)
**Influenza vaccines is costly and that is why it is not purchased normally**						
	65.77	84.23	81.17	59.38	80.36	86.43
**Side effects and safety concerns do not hinder physicians to get people vaccinated for influenza**						
	30.06	10.14	19.73	29.91	17.86	6.79
**Influenza vaccine prevents serious complications among patients with high-risk**						
	80.34	84.91	87.89	75.00	77.68	88.69
**I believe that mandatory flu vaccination of healthcare professionals will prevent influenza spread**						
	84.52	86.94	85.20	84.82	84.82	88.24
**I believe I can play a key role in the vaccination of my patients**						
	87.80	85.14	84.75	87.50	86.61	86.43
**Myself or my staff have participated in any training or continuing education related to the influenza vaccine in the past 12 months**						
	64.58	60.59	52.02	61.16	55.36	^#^77.38 (hij)
**Myself or my staff would be interested in participating in the trainings related to influenza vaccine**						
	^#^88.10 (g)	77.25	70.85	^#^85.27 (h)	^#^86.61 (h)	^#^87.33 (h)

*CL, confidence level; CPs, consulting physicians; NA, not applicable; OBGYN, obstetricians and gynecologists; Sig, significance*.

*Each category was assigned alphabets a–k, and if a category was significantly different from any other category, it was marked by the respective category alphabet in parentheses*.

# *Denotes statistically significantly higher proportion than the other respective categories mentioned in parenthesis. P values < 0.05 was considered as statistically significant*.

**Figure 2:**
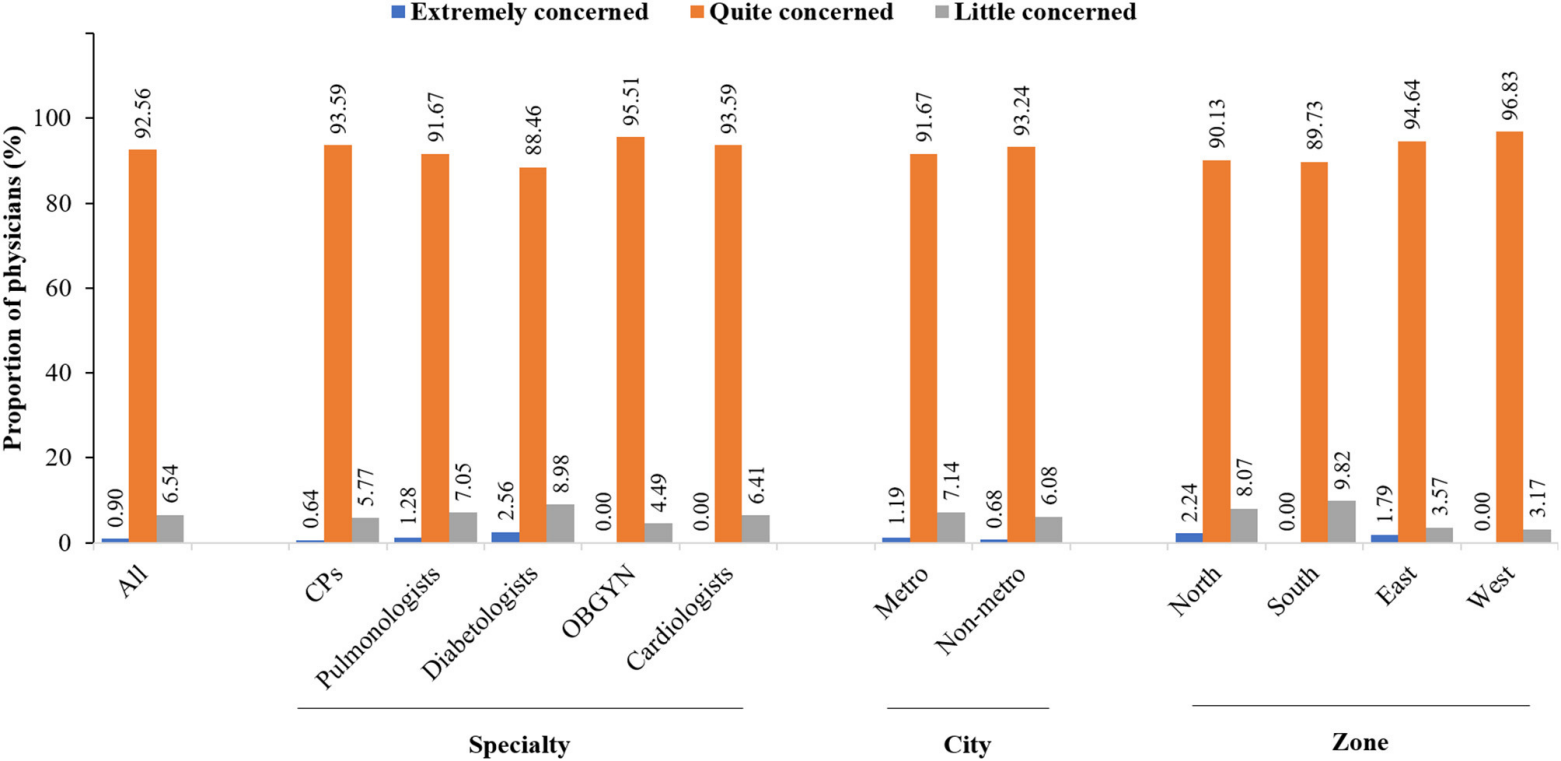
Overall attitude of physicians. CPs, consulting physician; OBGYN, obstetricians and gynecologists.

With regard overall attitude, majority (92.56%) of the physicians were “quite concerned,” whereas the remaining physicians were either a “little concerned” (6.54%) or “extremely concerned” (0.90%) toward influenza vaccination of the high-risk groups (Figure 2). There were no statistically significant differences in the attitude of physicians across different specialties, city of practice, and zones of practice.

### Physicians' Current Practices Related to Influenza Vaccination

Majority (82.82%) of the physicians responded that they offered influenza vaccine to patients in their routine clinical practice, but 41.41% of the physicians vaccinated <10% patients per month. More than 35% of the physicians required and offered the influenza vaccine (38.85%) or encouraged and offered the influenza vaccine (36.54%) to their office staff. There was no statistically significant difference between the proportion of physicians who provided the influenza vaccine in their clinical practice or those who required and offered the influenza vaccine to their office staff across different specialties. There was a statistically significant higher proportion of pulmonologists who vaccinated 25–40% of patients per month in comparison to diabetologists, obstetricians, gynecologists, and cardiologists. A significantly higher proportion of physicians in metro cities offered influenza vaccines to the patients, vaccinated 25 to >40% of patients per month, and encouraged and offered influenza vaccine to their office staff, in comparison to the physicians based in non-metro cities. A higher proportion of physicians in the west than those in north and south zones provided the influenza vaccine in their clinical practice. Additionally, a significantly higher proportion of physicians from the west also offered the vaccine to 10–40% of patients per month and required and offered the vaccine to their office staff in comparison to the physicians from north, south, and east zones (Table 4).

**Table 4:** Current physician practices toward influenza vaccine administration.

**Correct response, %**	**Overall** ***N* = 780**	**Specialty**				
		**CPs**	**Pulmonologists**	**Diabetologists**	**OBGYN**	**Cardiologists**
		**(*n* = 156)**	**(*n* = 156)**	**(*n* = 156)**	**(*n* = 156)**	**(*n* = 156)**
**Sig (95%CL)**		**a**	**b**	**c**	**d**	**e**
**Do you offer influenza vaccines?**						
Yes	82.82	78.21	83.33	85.26	82.05	85.26
No	17.18	21.79	16.67	14.74	17.95	14.74
**Percentage of overall patients vaccinated by the physician per month**						
<10	41.41	39.10	30.13	^#^41.03 (b)	^#^46.79 (ab)	^#^50.00 (ab)
10–25	32.69	32.69	35.90	37.82	29.49	27.56
25–40	19.10	23.72	^#^26.28 (cde)	11.54	18.59	15.38
>40	6.79	4.49	7.69	9.62	5.13	7.05
**Practices followed regarding influenza vaccine for office staff**						
We require and offer the influenza vaccine						
	38.85	41.67	41.67	35.90	41.03	33.98
We encourage and offer the influenza vaccine						
	36.54	^#^39.10 (d)	^#^39.74 (d)	^#^39.10 (d)	25.64	^#^39.10 (d)
We require, but do not offer, the influenza vaccine						
	13.59	8.33	10.90	^#^16.67 (a)	^#^18.59 (a)	^#^13.46
We encourage, but do not offer, the influenza vaccine						
	11.02	10.90	7.69	8.33	^#^14.74 (b)	13.46
**Correct response, %**	**City of practice**		**Zone of practice**			
	**Metro**	**Non-metro**	**North**	**South**	**East**	**West**
	**(** ***n*** **= 336)**	**(** ***n*** **= 444)**	**(** ***n*** **= 223)**	**(** ***n*** **= 224)**	**(** ***n*** **= 112)**	**(** ***n*** **= 221)**
**Sig (95%CL)**	**f**	**g**	**h**	**i**	**j**	**k**
**Do you offer influenza vaccines?**						
Yes	^#^86.01 (g)	80.41	78.03	81.70	83.04	^#^88.69 (hi)
No	13.99	^#^19.59 (f)	^#^21.97 (k)	^#^18.30 (k)	16.96	11.31
**Percentage of overall patients vaccinated by the physician per month**						
<10	29.17	^#^50.68 (f)	^#^60.09 (ijk)	31.25	^#^41.07 (i)	33.03
10–25	38.39	28.38	18.39	^#^45.54 (h)	^#^36.61 (h)	32.13 (h)
25–40	21.43 (g)	17.34	12.11	^#^14.29 (h)	13.90	^#^35.20 (hij)
>40	11.01 (g)	3.60	^#^9.42 (k)	^#^8.93 (k)	^#^8.04 (k)	1.36
**Practices followed regarding influenza vaccine for office staff**						
We require and offer the influenza vaccine						
	34.52	^#^42.12 (f)	14.35	^#^36.61 (hj)	20.53	^#^75.11 (hij)
We encourage and offer the influenza vaccine						
	^#^49.41 (g)	26.80	^#^40.81 (k)	^#^40.18 (k)	^#^55.36 (hik)	19.01
We require, but do not offer, the influenza vaccine						
	8.33	^#^17.57 (f)	^#^30.04 (ijk)	^#^11.16 (k)	^#^8.93 (k)	1.81
We encourage, but do not offer, the influenza vaccine						
	7.74	^#^13.51 (f)	^#^14.80 (k)	^#^12.05 (k)	^#^15.18 (k)	4.07

*CL, confidence level; CP, consulting physicians; OBGYN, obstetricians and gynecologists; Sig, significance*.

*Each category was assigned alphabets a–k, and if a category was significantly different from any other category, it was marked by the respective category alphabet in parentheses*.

# *Denotes statistically significantly higher proportion than the other respective categories mentioned in parenthesis. P value < 0.05 was considered as statistically significant*.

**Figure 3:**
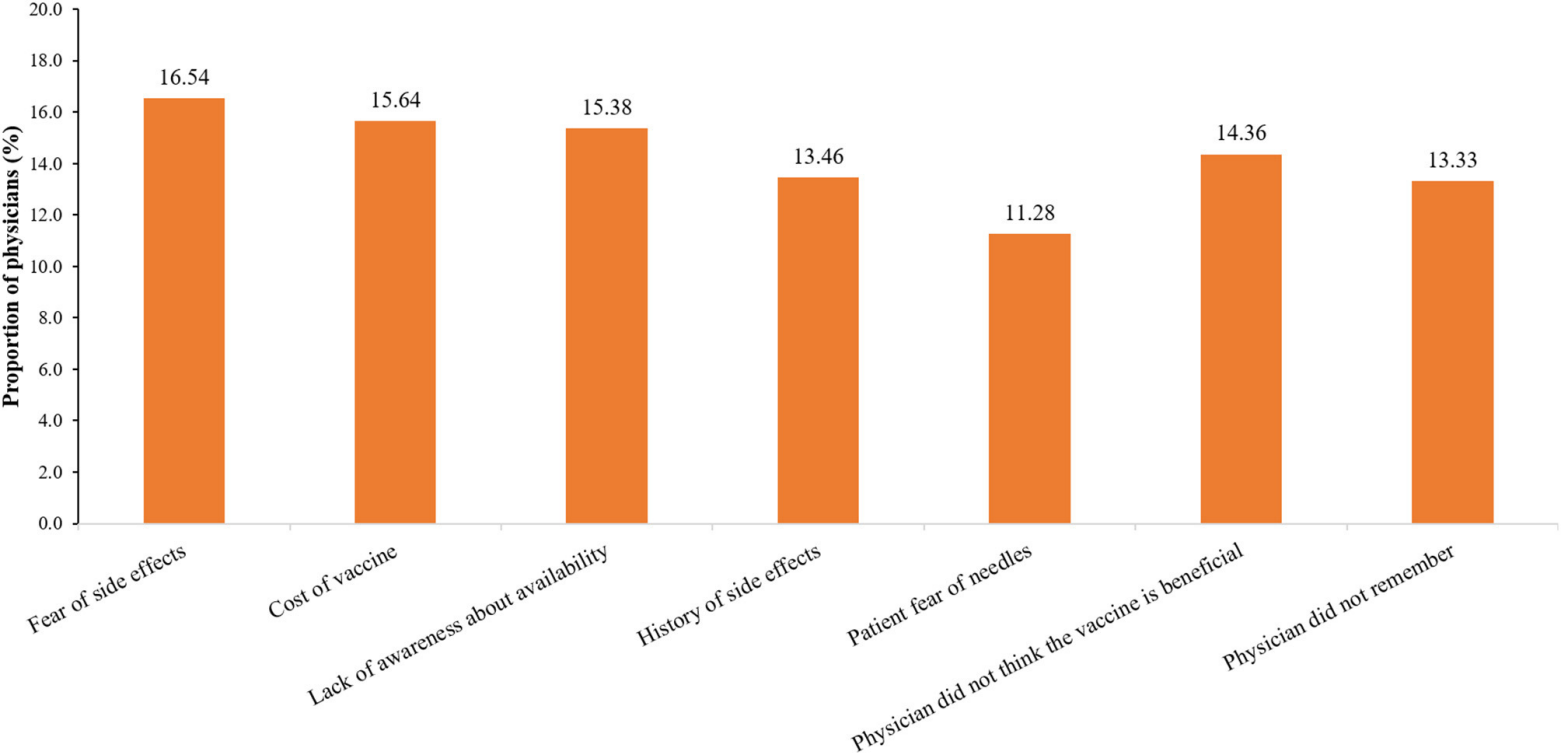
Physicians' common reasons for not prescribing influenza vaccine to patients. CPs, consulting physician; OBGYN, obstetricians and gynecologists.

Physicians provided various reasons for not prescribing the influenza vaccine to patients, including fear of side effects (16.54%), cost of the vaccine (15.64%), lack of awareness about availability (15.38%), lack of belief that the vaccine was beneficial (14.36%), history of side effects (13.46%), physicians did not remember (13.33%), and patient fear of needles (11.28%; Figure 3). There was no statistically significant difference between the physicians across different specialties, city of practice, and zone of practice in terms of reasons for not prescribing the influenza vaccine to the patients, except in the north zone where a significantly higher proportion of physicians did not think that the vaccine was beneficial.

## Discussion

The present study is the first-of-its kind to evaluate physicians' awareness, attitude, and current practices toward influenza vaccination across 14 cities from four zones in India. Majority of the physicians had a medium level of awareness and a “quite concerned” attitude toward influenza vaccination of high-risk groups, which was further reflected by the high frequency of providing the influenza vaccine to patients in routine clinical practice. However, some physicians did not prescribe the influenza vaccine to their patients for various reasons, of which their fear of side effects was predominant reason. There was a comparable level of awareness (medium/high) and attitude toward influenza vaccination among physicians from different specialties based at metro/non-metro cities in different zones of the country, which indicates a similar index of physicians' awareness and attitude across India. However, pulmonologists, physicians in metro cities, and those from the west zone had better clinical practices than physicians from other specialties, non-metro cities, and north/south/east zones.

Various studies have reported low awareness about influenza and influenza vaccination among HCPs due to their insufficient knowledge about the disease and a wide range of misconceptions (15, 17, 18, 24–26), all of which reduce the rate of self-vaccination and contribute to the vicious cycle of transmission of virus particles from HCPs to patients and other staff members. Hence, increased awareness about influenza vaccination among HCPs would play an important role in self-vaccination, prevention of iatrogenic and nosocomial infection, and promotion of effective vaccination among the general population.

The current survey found that the physicians were aware that they were at risk of getting influenza and should be vaccinated annually, had an understanding that they play an imperative role in disease transmission, and mandatory vaccination could prevent the spread of influenza to others. A recent study suggests that approximately two-thirds of Indians are not aware of adult vaccination (27). Here, HCPs can serve as advocates of adult vaccination and convince the general population about the role and benefits of vaccination (24).

Annual influenza vaccination is recommended for all high-risk groups given their susceptibility to influenza-related complications and mortality (5, 28). In our survey, most of the physicians were aware that all the high-risk groups have higher susceptibility and that vaccination could help prevent severe complications. Physicians also had increased awareness about CDC recommendations on influenza shots for HCPs, the guideline(s) on preventive care for influenza, annual vaccination, and before the start of flu season as the appropriate time for vaccination.

Majority of the physicians were also aware of signs and symptoms of the disease, of coughing and sneezing as the primary mode of transmission of the virus, and <8–10 days post-exposure to influenza virus as the time of symptom appearance. They were also aware about influenza vaccines being live or attenuated, differences between TIVs and QIVs, the broader protection of QIVs over TIVs, differences between subunit and split vaccines, the lower reactogenicity of subunit vaccines, tolerability of influenza vaccines, and reduction in efficacy because of mismatch of virus strains. Similar results regarding physicians' awareness about influenza and influenza vaccination were reported in previous studies (19, 20).

The influenza virus is transmitted from infected persons to their close contacts even in the absence of clinical symptoms, i.e., during the asymptomatic stage, *via* viral shedding (though lasting for a shorter duration) (29, 30). Various factors determine transmission by asymptomatic patients, including their proportion, infectiousness, and the host and immune factors in their close contacts (30). Therefore, patients and HCPs should be vaccinated before the start of the influenza season, and HCPs should take all preventive measures like the use of masks and proper sanitization practices during the influenza season to reduce the transmission rate of influenza virus from asymptomatic patients. In our study, only 37.56% of the physicians were aware that asymptomatic patients could also transmit the disease. This result was in agreement with that of another study where 32.4% of HCPs mentioned that asymptomatic patients could transmit the disease (31). In contrast, a study by Iftikhar et al. reported a higher level of awareness among physicians (61.9%) regarding the transmission of disease by asymptomatic patients (19). Hence, training sessions on the etiology and pathogenesis of influenza virus infection and influenza virus statistics/burden in the country should be scheduled to increase HCPs awareness on influenza infection.

In the current study, the vaccination rate of physicians in last 6 to 12 months was 57.95%, which was comparatively higher than that reported in previous studies (19, 32), indicating an improvement in physician attitude about influenza vaccination in recent years. Cost-effectiveness is crucial to the acceptance of any vaccine in the general population. In a recent survey conducted by Sundaram et al., 93% of the participants accepted influenza vaccination at no cost (33). In our study as well, 76.28% of the physicians believed that patients did not purchase the influenza vaccine due to its high cost. However, physician advice has always been considered as the most imperative method for educating patients regarding the risks of influenza-related illness and the benefits of influenza vaccination and for encouraging them to get vaccinated for influenza at least once a year (22, 34, 35). In a recent Indian study, physicians' recommendations were more effective in increasing the acceptance level of vaccination in comparison to reduction in vaccine cost (36). Similar results were also seen in the current study where majority of the physicians considered their word of mouth, in-clinic patient education, and public awareness campaigns as effective ways of publicizing influenza vaccination. Another study also reported face-to-face interactions, posters, brochures, text reminders, telephone calls, and email reminders as the preferred methods to promote vaccination to the patients (20).

Most physicians believed that they could play a key role in vaccination of their patients and had also offered vaccination to their patients; this might be due to increased awareness of patients about influenza vaccination during coronavirus disease 2019 (COVID-19) pandemic. They also considered influenza vaccination to be a part of their routine medical practice. Our results were in concordance with the previously published study results (20). However, some physicians did not prescribe influenza vaccine to their patients due to fear of side effects, cost of the vaccine, lack of aware about availability, lack of belief that the vaccine is beneficial, history of side effects, or due to patient fear of needles. Similar barriers were identified in other studies as well (19, 20, 26, 37–41). In a meta-analysis of 21 studies, the top five categories for refusing the influenza vaccine were fear of adverse reactions, lack of concern, inconvenient delivery, lack of perception of own risk, and doubts about vaccine efficacy (18).

In India, influenza vaccination continues to remain an underutilized opportunity toward reducing the burden of preventable diseases owing to the presence of certain misconceptions among physicians. Adult immunization is negligible in India due to the lack of surveillance, nationally adopted adult immunization guidelines, and coordinated adult immunization programs, missed opportunities for vaccination, cost, lack of provider recommendation, lack of knowledge or recognition of benefits/efficacy/safety, lack of infrastructure, or vaccine hesitancy (24, 42, 43). To overcome these barriers, government aids should be provided for mandatory influenza vaccination of HCPs; for (1) educational or promotional programs on the benefits and misconceptions related to vaccination *via* campaigns, and print and digital media platforms; (2) introduction of coordinated adult immunization programs; (3) incorporation of adult vaccination into regular check-ups and provision of the vaccine at the subsidized cost; and (4) promotion of life-course immunization.

Our study has a few strengths and limitations. This study is the first-of-its-kind in India to evaluate the awareness, attitude, and current practices of a large pool of physicians toward influenza and influenza vaccination. It was a pan India study covering physicians across five specialties, 14 cities, and four zones, and an elaborate questionnaire encompassing different assessment areas. Exclusion of specialists other than consulting physicians, pulmonologists, diabetologists, cardiologists, and obstetricians/gynecologists and influence of recall bias on survey outcomes were the key limitations of this study. Furthermore, no reliability statistics was done in the study and no association was determined for awareness, attitude, and practices with background characteristics of the study participants. As the data are limited to the Indian context, generalization of the study results to other geographies was not possible.

## Conclusion

In conclusion, in this first of its kind pan-India survey on influenza infection and vaccination, more than half the participating physicians showed medium level of awareness and this differed across specialties. Majority of the physicians showed strong attitude toward influenza vaccination of high-risk groups and offered the influenza vaccine to their patients. Fear of side effects, cost, lack of awareness about availability, lack of belief that the vaccine is beneficial, history of side effects, or patients' fear of needles were some of the main reasons for not prescribing the vaccine. Educational strategy implementation was highlighted as a key area for the future to improve the overall understanding and practices of physicians toward influenza vaccination especially in high-risk groups.

## Data Availability Statement

The raw data supporting the conclusions of this article will be made available by the authors, without undue reservation.

## Ethics Statement

Ethical review and approval was not required for the study on human participants in accordance with the local legislation and institutional requirements. The patients/participants provided their written informed consent to participate in this study.

## Author Contributions

Both authors met the International Council of Medical Journal Editors' criteria for authorship and participated in the design, implementation, analysis, interpretation of the study, and provided the final approval of the manuscript. Both authors had full access to all the data in the study and had a final responsibility for the decision to submit for publication. Both authors contributed to the article and approved the submitted version.

## Conflict of Interest

AS is an employee of Abbott India Limited. The remaining author declares that the research was conducted in the absence of any commercial or financial relationships that could be construed as a potential conflict of interest.

## Publisher's Note

All claims expressed in this article are solely those of the authors and do not necessarily represent those of their affiliated organizations, or those of the publisher, the editors and the reviewers. Any product that may be evaluated in this article, or claim that may be made by its manufacturer, is not guaranteed or endorsed by the publisher.
