# Supplementary material for: Awareness, Attitude, and Current Practices Toward Influenza Vaccination Among Physicians in India: A Multicenter, Cross-Sectional Study
Source: Front Public Health. 2021 Aug 23;9:642636. doi: 10.3389/fpubh.2021.642636 (PMC8419342; doi:10.3389/fpubh.2021.642636)
Supplement: Supplementary file 1 [file Table_1.DOCX]

**Supplementary Table 1.** **Survey questionnaire**

| **SECTION 1: AWARENESS ABOUT INFLUENZA AND INFLUENZA VACCINATION** | | | | | | | | | | |
| --- | --- | --- | --- | --- | --- | --- | --- | --- | --- | --- |
| **Q1. Influenza is more serious than a “common cold”** | | | | | | | | | | |
| Correct | | | | Incorrect | | | | Not sure | | |
| **Q2. The signs and symptoms of influenza include fever, headache, sore throat, cough, nasal congestion, and aches and pains** | | | | | | | | | | |
| Correct | | | | Incorrect | | | | Not sure | | |
| **Q3. Adults with influenza commonly experience nausea and vomiting or diarrhea** | | | | | | | | | | |
| Correct | | | | Incorrect | | | | Not sure | | |
| **Q4. Symptoms typically appear 8 to 10 days after a person is exposed to influenza** | | | | | | | | | | |
| Correct | | | | Incorrect | | | | Not sure | | |
| **Q5. Influenza is transmitted primarily by coughing and sneezing** | | | | | | | | | | |
| Correct | | | | Incorrect | | | | Not sure | | |
| **Q6. Influenza is transmitted primarily by contact with blood and body fluids** | | | | | | | | | | |
| Correct | | | | Incorrect | | | | Not sure | | |
| **Q7. People with influenza can transmit the infection only after their symptoms appear** | | | | | | | | | | |
| Correct | | | | Incorrect | | | | Not sure | | |
| **Q8. Not everyone in the general public is familiar with influenza vaccination** | | | | | | | | | | |
| Strongly agree | Agree | | | | Do not know | | Disagree | | | Strongly disagree |
| **Q9. What do you think is the most effective way of publicizing influenza vaccine? (you may state multiple answers for this question)** | | | | | | | | | | |
| Posters/information leaflets | | | Healthcare physician by his/her word of mouth | | | | | In-clinic patient education/counselling | | |
| Public awareness campaigns | | | Digital medium | | | | | Patient support groups | | |
| **Q10. Influenza vaccines can be live or inactivated** | | | | | | | | | | |
| True | | | | | | False | | | | |
| **Q11. In case of mismatch of virus strains, the influenza vaccine efficacy may be reduced** | | | | | | | | | | |
| Correct | | | | Incorrect | | | | Not sure | | |
| **Q12. I believe influenza vaccine is tolerable** | | | | | | | | | | |
| Yes | | | | No | | | | Can’t say | | |
| **Q13. The inactivated influenza vaccine contains live viruses that may cause some people to get influenza** | | | | | | | | | | |
| Correct | | | | Incorrect | | | | Not sure | | |
| **Q14. How often do you think the influenza vaccine should be administered?** | | | | | | | | | | |
| Every 6 months | | Every year | | | | Every 5 years | | | Once in a lifetime | |
| **Q15. How long do you think influenza vaccine can protect?** | | | | | | | | | | |
| 6-8 months | | | | 1-2 years | | | | Lifetime | | |
| **Q16. What is the appropriate time to give influenza vaccine?** | | | | | | | | | | |
| Before flu season starts | | | | During the flu season | | | | Immediately after the flu season | | |
| **Q17. As per your opinion, which are the high-risk groups associated with influenza?** | | | | | | | | | | |
| Pregnancy | | Children | | | | Elderly | | | All of the above | |
| **Q18. Influenza vaccine needs to be taken on an annual basis** | | | | | | | | | | |
| Correct | | | | Incorrect | | | | Not sure | | |
| **Q19. As a physician, do you feel you are at risk to get influenza and should get vaccinated annually?** | | | | | | | | | | |
| Yes | | | | No | | | | Not sure | | |
| **Q20. Can physicians spread influenza to their patients?** | | | | | | | | | | |
| Yes | | | | No | | | | Not sure | | |
| **Q21. Which is the guideline on preventive care for influenza?** | | | | | | | | | | |
| Name of guideline: _________________________________ | | | | | | No guideline | | | | |
| **Q22. Do you believe that the Center for Disease Control (CDC) recommends that health care practitioners should receive the flu shot?** | | | | | | | | | | |
| Yes | | | | No | | | | Not sure | | |
| **Q23. Do you know the difference between trivalent and quadrivalent influenza vaccines?** | | | | | | | | | | |
| Yes | | | | No | | | | Not sure | | |
| **Q24. A quadrivalent flu vaccine offers broader protection over a trivalent flu vaccine** | | | | | | | | | | |
| Yes | | | | | | No | | | | |
| **Q25. Do you know the difference between subunit and split influenza vaccines?** | | | | | | | | | | |
| Yes | | | | No | | | | Not sure | | |
| **Q26. A subunit flu vaccine is less reactogenic** | | | | | | | | | | |
| Yes | | | | | | No | | | | |
| **SECTION 2: ATTITUDE TOWARD INFLUENZA VACCINATION** | | | | | | | | | | |
| **Q1. In last 6-12 months, have you vaccinated yourself against influenza?** | | | | | | | | | | |
| Yes | | | | No | | | | Never vaccinated in last 2-3 years | | |
| **Q2. Do you think administering the influenza vaccine should be part of your routine medical practice?** | | | | | | | | | | |
| Yes | | | | No | | | | Can’t say | | |
| **Q3. Influenza vaccines is costly that’s why not purchased normally** | | | | | | | | | | |
| Strongly agree | Agree | | | | Do not know | | Disagree | | | Strongly disagree |
| **Q4. Side effects and safety concerns hinder physicians to get people vaccinated for influenza** | | | | | | | | | | |
| Strongly agree | Agree | | | | Do not know | | Disagree | | | Strongly disagree |
| **Q5. Influenza vaccine prevents serious complications of an influenza infection among patients with high-risk** | | | | | | | | | | |
| Strongly agree | Agree | | | | Do not know | | Disagree | | | Strongly disagree |
| **Q6. I believe that mandatory flu vaccination of health care professionals will prevent influenza spread** | | | | | | | | | | |
| Yes | | | | No | | | | Can’t say | | |
| **Q7. I believe I can play a key role in the vaccination of my patients** | | | | | | | | | | |
| Yes | | | | No | | | | Can’t say | | |
| **Q8. Have you or your staff participated in any training or continuing education related to the influenza vaccine in the past 12 months?** | | | | | | | | | | |
| Yes | | | | | | No | | | | |
| **Q9. Would you or your staff be interested in participating in the trainings related to influenza vaccine?** | | | | | | | | | | |
| Yes | | | | | | No | | | | |
| **SECTION 3. CURRENT PHYSICIAN PRACTICES** | | | | | | | | | | |
| **Q1. Do you offer the influenza vaccine?** | | | | | | | | | | |
| Yes | | | | | | No | | | | |
| **Q2. If you offer the influenza vaccines, what is percentage of patients in your clinic/hospital who are provided with influenza vaccination in a month by you?** | | | | | | | | | | |
| Less than 10 | | 10-25 | | | | 25-40 | | | More than 40 | |
| **Q3. Why did you not prescribe influenza vaccine to your patients? (you may state multiple answers for this question)** | | | | | | | | | | |
| Fear of side effects | | | | Past history of side effects | | | | Patient scared of needles | | |
| Cost of vaccine | | | | Not aware of the availability | | | | Did not remember | | |
| Did not think it is beneficial | | | | Other reason | | | | Not applicable | | |
| **Q4.Which practice do you follow regarding influenza vaccine for office staff?** | | | | | | | | | | |
| We require and offer the influenza vaccine | | | | | | We encourage and offer the influenza vaccine | | | | |
| We require, but do not offer, the influenza vaccine | | | | | | We encourage, but do not offer, the influenza vaccine | | | | |
